# [Retracted] MicroRNA‑1297 contributes to tumor growth of human breast cancer by targeting PTEN/PI3K/AKT signaling

**DOI:** 10.3892/or.2023.8617

**Published:** 2023-08-21

**Authors:** Chao Liu, Zhikui Liu, Xiao Li, Xiaojiang Tang, Jianjun He, Shaoying Lu

Oncol Rep 38: 2435–2443, 2017; DOI: 10.3892/or.2017.5884

Following the publication of this paper, it was drawn to the Editor's attention by a concerned reader that the tumour images shown in Fig. 7A and certain of the cell proliferation assay images shown in Fig. 3B were strikingly similar to data that had already appeared in another article written by different authors at different research institutes [Xiao W Wang, J, Li H, Xia D, Yu G, Yao W, Yang Y, Xiao H, Lang B, Ma X *et al*: Fibulin-1 is epigenetically down-regulated and related with bladder cancer recurrence. BMC Cancer 14: 677, 2014].

Owing to the fact that the contentious data in the above article had already been published prior to its submission to *Oncology Reports*, the Editor has decided that this paper should be retracted from the Journal. The authors were asked for an explanation to account for these concerns, but the Editorial Office did not receive a reply. The Editor apologizes to the readership for any inconvenience caused.

